# Identification of *VPS35* p.D620N mutation-related Parkinson’s disease in a Taiwanese family with successful bilateral subthalamic nucleus deep brain stimulation: a case report and literature review

**DOI:** 10.1186/s12883-017-0972-5

**Published:** 2017-10-06

**Authors:** Ying-Fa Chen, Yung-Yee Chang, Min-Yu Lan, Pei-Lung Chen, Chin-Hsien Lin

**Affiliations:** 1 Department of Neurology, Kaohsiung Chang Gung Memorial Hospital, Kaohsiung, Taiwan; 2 Center for Parkinson’s Disease, Kaohsiung Chang Gung Memorial Hospital, Kaohsiung, Taiwan; 3 Department of Medical Genetics, National Taiwan University Hospital, Taipei, Taiwan; 4 Graduate Institute of Medical Genomics and Proteomics, National Taiwan University College of Medicine, Taipei, Taiwan; 5 Department of Neurology, National Taiwan University Hospital, Taipei, 100 Taiwan

**Keywords:** Parkinson’s disease, VPS35, Mutation, Deep brain stimulation

## Abstract

**Background:**

Vacuolar protein sorting 35 (*VPS35*) was recently reported to be a genetic cause for late-onset autosomal dominant Parkinson’s disease (PD). However, *VPS35* mutations are rarely reported in Asian populations. Herein, we report the first Taiwanese family with the pathogenic *VPS35* p.D620N mutation, including one patient treated successfully with subthalamic nucleus deep brain stimulation (STN-DBS).

**Case presentation:**

A 61-year-old woman presented with progressive left hand resting tremor at the age of 42. Neurological examinations revealed mask face and akinetic-rigidity over left extremities. She showed a good response to levodopa treatment, and her unified Parkinson’s disease rating scale (UPDRS) motor scores improved from 42 to 15 under the levodopa equivalent dose of 1435 mg/day. She developed peak-dose dyskinesia and motor fluctuation seven years after the onset of symptoms, and received bilateral STN-DBS at the age of 55. Stimulation led to a marked improvement in her motor symptoms with a 37% improvement in the UPDRS motor score during the OFF period five years after surgery. The patient’s mother and three siblings were also diagnosed with PD in their forties, following an autosomal-dominant inheritance pattern. We performed genetic analysis of the proband using a targeted next generation sequencing (NGS) panel covering 17 known PD-causative genes. We identified a pathogenic missense mutation in *VPS35* gene, c.1858G > A (p.D620N), in this patient.

**Conclusions:**

This is the first report of the *VPS35* p.D620N mutation in a Taiwanese family. Additionally, our report contributes to the current understanding of genetically defined PD patients treated successfully with STN-DBS.

## Background

Parkinson’s disease (PD) is a common neurodegenerative disorder that affects more than 1% of the global population over the age of 60 [1]. Mutations in a number of pathogenic genes have been identified over the last two decades and found to be associated with both familial and sporadic PD [2]. Recent advances in next-generation sequencing (NGS) technology have rapidly facilitated the identification of novel genes causing PD. This genetic information can lead to a better understanding of disease-associated protein networks as well as provide key biological insights into underlying disease processes of PD, including protein degradation dysregulation, mitochondrial dysfunction, and abnormal trafficking of vesicles and proteins [3].

Vacuolar protein sorting 35 (*VPS35*) was recently identified to be a pathogenic gene for late-onset autosomal dominant PD by whole exome sequencing [4, 5]. VPS35 is a key component of the retromer cargo-recognition complex, known to regulate the process of protein sorting between tubular endosomes and the trans-Golgi network [6]. Pathogenic mutations of *VPS35* could cause a disruption of the retrograde intracellular protein transport system and contribute to neuronal degeneration in PD. One missense mutation, c.1858G > A (p.D620N), has been reported to be pathogenic in patients with autosomal-dominant PD in several European and American families [4, 5, 7]. Although many populations were screened for *VPS35* mutations, few Asian patients were identified to carry the *VPS35* p.D620N mutation [4, 8, 9].

Here we report the first Taiwanese family with the pathogenic *VPS35* p.D620N mutation, including one patient treated successfully with subthalamic nucleus deep brain stimulation (STN-DBS). We compare the clinical features of the mutation carriers in the index family with the characteristics of previously reported *VPS35* p.D620N patients derived from a literature review.

## Case presentation

**Fig. 1:**
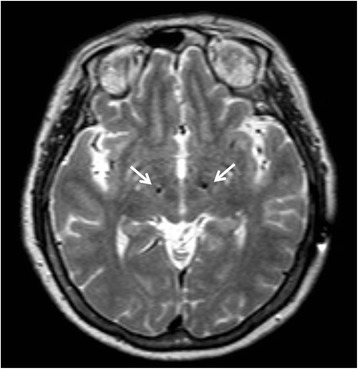
Post-surgery Brain MRI of the index patient. Arrows indicate the implanted locations of leads of DBS

A 61-year-old woman with no underlying medical conditions presented with progressive left hand resting tremor that began at the age of 42, quickly followed by the development of rigidity and slow movement over the left side limbs. There were no signs of early cognition impairment, change in personality, behavior disturbance, psychiatric symptoms, limited eye movements, bulbar symptoms, retrocollis or antecollis, focal weakness, focal numbness, or gait disturbance. Treatment with levodopa, anticholinergics and dopamine agonists led to a significant clinical improvement. The Unified Parkinson’s Disease Rating Scale (UPDRS) motor score improved from at 42/108 to 15/108 after medications. However she developed peak-dose dyskinesia and wearing-off phenomenon within seven years, which clinical responses became progressively resistant to medications. She developed severe gait freezing, dysphagia and torticollis during OFF-medication status. She then received STN-DBS at the age of 55. The levodopa equivalent daily dose was 1435 mg/day before the DBS surgery. Monopolar stimulation of the two dorsal contacts (6−/2-) at 4.4 V in the right STN and 3.2 V in the left STN, with a frequency of 160 Hz and pulse duration of 90 ms in the right STN and 60 ms in the left STN led to the best clinical response. We measured the intraoperative microelectrode recording (MER) in STN during surgery. MER could identify the neuronal firing patterns of the STN and is the most commonly employed technique to assist and validate target localization. The recorded MER of our index patient was comparable with those with sporadic PD. The post-operational brain MRI was shown in Fig. 1. Stimulation resulted in a marked improvement in her parkinsonism features and decrease of peak-dose dyskinesia. STN-DBS still resulted in a 37% improvement in the UPDRS motor score during the OFF period five years after surgery. The observed motor benefit persisted, with UPDRS motor scores of 35/108 in the OFF-medication condition and OFF-stimulation, 22/108 in the OFF-medication and ON-stimulation condition, 15/108 in the ON-medication and OFF-stimulation condition, and 13/108 in the ON-medication and ON-stimulation condition.

**Fig. 2:**
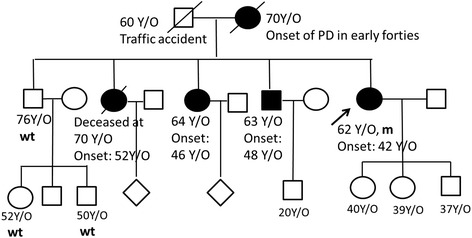
Family pedigree and genetic analysis of the index patient. Index family pedigree. Black symbols denote family members affected with PD. The proband described in the current study is marked with an arrow. m, mutated alleles; wt, wild type alleles

The patient’s family history indicated that her deceased mother, two sisters (one deceased) and one brother were also diagnosed with PD in their mid to late forties (Fig. 2). Unfortunately, the affected sister and brother declined genetic analysis and detailed examination. Neurological examinations of the other family members were normal, including additional siblings and the younger generation. Since the clinical picture indicated an autosomal dominant inheritance form of PD, we applied targeted NGS to perform genetic analysis of the proband, covering candidate genes known causing familial forms of PD [10]. Variants within the genes of interest were extracted for further analysis and included *SNCA, GBA, LRRK2, UCHL1, GIGYF2, VPS35, DNAJC13, PARK2, PINK1, DJ-1,ATP13A2, HtrA2,FBXO7, SYNJ1, DNAJC6, CHCHD2,* and *Rab39B*. The analyzed genes presented an individual average coverage of >33× and we Sanger sequenced exons in specific samples with coverage below 8×. We assessed the frequency of the variants in the general population (Exome Aggregation Consortium (ExAC), dbSNP, 1000 Genomes Project) and Taiwan biobank (https://www.twbiobank.org.tw/new_web/index.php), which is the whole genome sequencing (WGS) database enrolling 997 Taiwanese people without known neurological disorders, in order to identify potentially pathogenic variants. If the variants were non-synonymous, predicted to change the protein sequence, or were located in either untranslated or splicing regions, we also evaluated the functional annotation of the variants and used prediction software to obtain a prediction of pathogenicity. We considered variants with minor allele frequency (MAF) < = 0.1% (rare variants) and variants predicted to change the protein sequence or to impact splicing as potentially pathogenic. Mutations were classified as definitely pathogenic if they had been reported in the literature previously as causative. The salsa multiplex ligation-dependent probe amplification (MPLA) kit P051-c1/P52-c1 (MRC-Holland, Amsterdam, The Netherlands) was used to detect large deletions or duplications of common PD-causative genes, including *SNCA*, *Parkin*, *PINK1*, *DJ-1*, *ATP13A2*, *PLA2G6*, *FBXO7*, *DNAJC6* and *LRRK2*.

**Fig. 3:**
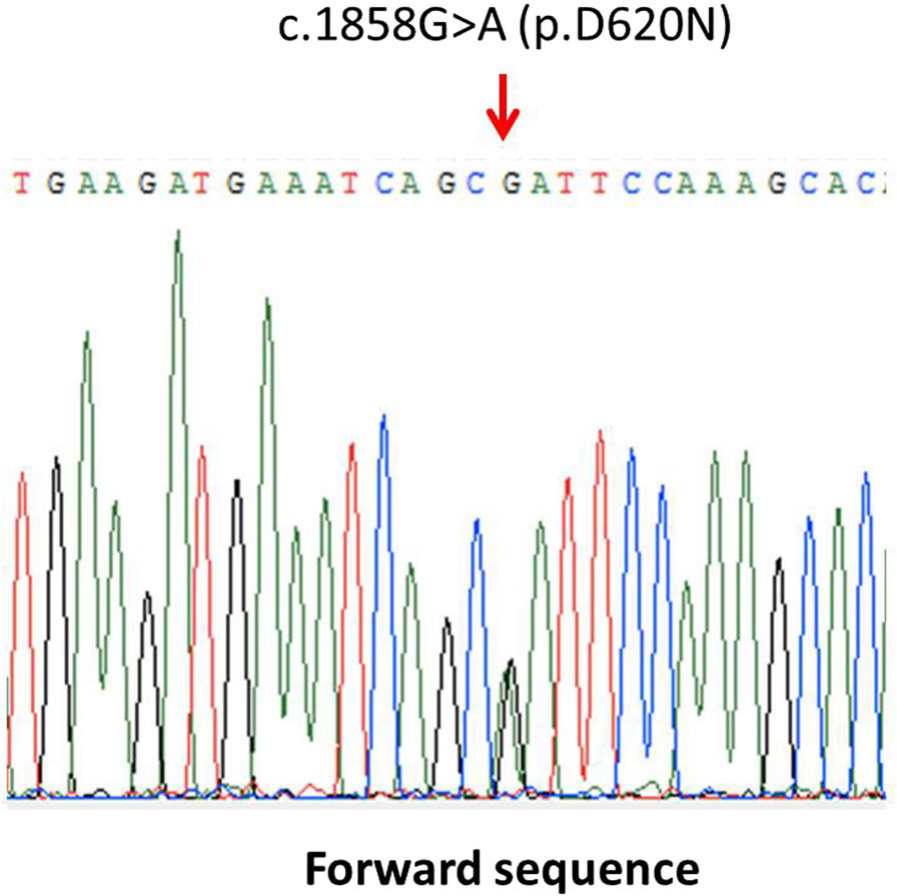
Chromatograms of direct sequencing of the *VPS35* genomic sequence. Genetic analysis reveals a single nucleotide change (c.1858G>A, p.D620N) by Sanger sequencing. The mutation identified in this study was located in the indicated position

We determined that this patient carried a heterozygous missense substitution in the *VPS35* gene, c.1858G > A (p.D620N) (Fig. 3). We did not identify other rare genetic variants from the targeted NGS sequencing panel. The results of MLPA did not detect any genetic deletions or duplications of *SNCA* and other aforementioned common PD-causative gene. This variation had been reported in previous studies to be the pathogenic for patients with autosomal dominant forms of PD, was not found in more than 500 Japanese control subjects [8] and was not identified from the WGS database of Taiwan Biobank. In the patient’s family this genetic substitution was not observed in the non-affected family members (Fig. 1), although the affected siblings declined genetic analysis which hampered further segregation analysis. The Institutional Ethics Committee of National Taiwan University Hospital approved this study and each subject that provided blood samples for genetic testing also provided written informed consent.

## Discussion and conclusions

*VPS35* was found to be a pathogenic gene for autosomal-dominant PD, and a single mutation, p.D620N, has been consistently reported in unrelated families originating primarily from western ancestry. Excluding one report showing 3 families with PD (1.0% of familial cases) and 1 sporadic PD patient (0.23% of sporadic PD) with the *VPS35* p.D620N mutation in a Japanese population, this mutation was previously found to be extremely rare in Asian populations, including Taiwanese and mainland Chinese populations (0%) [4, 9]. To our knowledge, this is the first report of a Taiwanese family carrying a pathogenic heterozygous *VPS35* p.D620N mutation presenting as an idiopathic PD phenotype and showing a long term positive response to bilateral STN-DBS.

Pathogenic mutations in the *VPS35* gene have been identified but as a rare genetic cause of autosomal dominant PD development. Mutation carriers, particularly carriers of the p.D620N missense mutation, are estimated to account for less than 1% of the entire PD population [4, 5, 8, 9, 11, 12]. The *VPS35* p.D620N mutation was identified in 3 Austrian families and a single family each in Switzerland, the United States, Tunisia, the United Kingdom and Japan, as well as one family and one patient with sporadic PD among Yemenite Jewish populations in Israel and Japan (summarized in Table 1). *VPS35* p.D620N mutation carriers were recently identified in a Japanese population with a mutation frequency of 1.0% for autosomal dominant forms of PD and 0.23% for sporadic PD patients. This observation inspired the hypothesis that the p.D620N substitution could be a mutational hot spot for autosomal dominant PD across populations [8]. Our findings reinforce this hypothesis and indicate that although previous genetic screening studies conducted in Chinese populations were negative [4, 9], *VPS35* p.D620N should be considered in patients with autosomal-dominant forms of PD in Taiwanese populations. Combining previous reports with our findings, we observe that the average age at symptoms onset for PD patients carrying the *VPS35* p.D620N mutation is 35–68 years (Summarized in Table 1), slightly but distinctively earlier onset age relative to idiopathic PD patients. In our family, the most notable feature was the relatively early age of onset, in the early forties. The clinical symptoms of *VPS35* p.D620N mutation carriers closely resemble that seen in idiopathic PD patients, and most carriers present with dopa-responsive parkinsonism (Summarized in Table 1). Onset was generally unilateral, with tremor-predominant symptoms and a slow course of progression. Psychiatric problems were not prominent, and only one patient was reported to have depression [13]. However, dementia was noted in patients with a long disease course [7], and two patients in the same British family showed mild to moderate olfactory dysfunction compared to controls from the same population [13]. Based on these findings, we conclude that patients with the *VPS35* mutation could show a comparatively benign disease course without prominent atypical features.

**Table 1:** Clinical phenotypes of patients carrying *VPD35* p.D620N mutation

Reference	Age of onset (years, range)	Tremor-predominant	Akinetic-rigidity-predominant	Responses to levodopa	Other features	Ethnicity
Vilarin˜o-Gu¨ell C et al., 2011 [4]	50.6 ± 7.3 (42–64)	+	–	Good	–	Swiss, American, Tunisian, Yemenite Jews
Sheerin UM et al., 2012 [7]	42.7 ± 5.1 (35–52)	+	+	Good	Olfactory dysfunction, dementia	British
Ando M et al., 2012 [8]	48.3 ± 10.3 (34–62)	+	–	Good	–	Japanese
Struhal W et al., 2014 [13]	50 (40–68)	+	+	Good	Depression	Australian
Current study	42–52	+	–	Good	Dementia	Taiwanese

The index patient reported here received bilateral STN-DBS 13 years after symptom onset, and she displayed a sustained motor benefit in response to the treatment. One previous study also reported the therapeutic responses of two patients carrying the *VPS35* p.D620N mutation and receiving STN-DBS [14], and both showed persistent benefit at 8 year follow-up. An additional two cases including a *VPS35* mutation and STN-DBS treatment have been reported in the literature previously [7, 15]. The first patient benefitted from STN-DBS treatment [7] but the second patient had little DBS benefit due to dysarthria [15]. The beneficial effects of STN-DBS were comparable in patients carrying *VPD35* p.D620N mutation and patients with sporadic PD (Table 2) [16]. The surgery was done 10 years after onset of motor symptoms in average and the percentage of motor symptom improvement, scored by part III of UPDRS, was more than 30% in both group one year after surgery. The decreased dosage of LEDD maintained 5 years after surgery both in patients with and without *VPD35* p.D620N mutation. The results presented here contribute to the current understanding of STN-DBS treatment for genetically defined PD patients. Subjects carrying a PD-associated mutation seem to receive as much benefit from DBS surgery as patients without an apparent genetic background, suggesting that the indication for surgery should be based on the disease phenotype rather than its genotype.

**Table 2:** Comparison of the therapeutic effects of DBS in patients carrying *VPD35* p.D620N mutation and sporadic PD

Reference	*VPD35* p.D620N mutation carriers	Age of onset (mean ± SD, years)	Age at receiving DBS (mean ± SD, years)	DBS stimulation details that gained the best clinical response	LEDD before DBS (mg)	LEDD one year after DBS (mg)	Improvement of UPDRS motor scores one year after DBS in “OFF” medications	LEDD five to eight years after DBS (mg)
Fleury V et al., 2013 [14]	+ (patient 1)	49	60	Monopolar stimulation of two most proximal contacts (3-) at 2.2 V in the right STN and 2.4 V in the left STN, with a frequency of 130 Hz and pulse duration of 60 ms.	1540	400	76%	550 (eight years after DBS)
	+ (patient 2)	45	55	Monopolar stimulation of the 3rd and 4th distal contact (2–3-) in the right STN at 3.3 V and 3.0 V in the left STN, with a frequency of 180 Hz and pulse duration of 90 ms.	1276.5	300	36%	N.A.
Current study	+ (index patient)	42	55	Monopolar stimulation of the two dorsal contacts (6−/2-) at 4.4 V in the right STN and 3.2 V in the left STN, with a frequency of 160 Hz and pulse duration of 90 ms in the right STN and 60 ms in the left STN	1435	1000	37%	1160 (five years after DBS)
Aviles-Olmos I et al., 2014 [16]	- 41 sporadic PD patients	43.3 ± 9.8	56.2 ± 8.4	Monopolar stimulation	1471 ± 515	949 ± 572	45.5 ± 12.8%	668 ± 359 (five years after DBS)

*DBS* deep brain stimulation, *STN* subthalamic nucleus, *LEDD* levodopa equivalent dose, *SD* standard deviation, *N.A.* not available

This report has several strengths, based on the fact that is the first report of a family carrying a pathogenic *VPS35* p.D620N mutation in a Taiwanese population, reinforcing the role of *VPS35* mutations in PD pathogenesis [4–6]. Mutations in *VPS35* may cause disruption of intracellular trafficking and lead to neurodegeneration, similarly to α-synuclein and Lrrk2, also known to be involved in vesicle and protein trafficking [17, 18]. There are also some limitations in the current study, including the limited number of enrolled family members and the fact that some affected family members declined genetic analysis.

In conclusion, we present a report of the first Taiwanese family carrying the pathogenic *VPS35* p.D620N mutation. These findings suggest that the *VPS35* mutation should be considered in patients with an autosomal-dominant family history, relatively young onset, and tremor predominant symptoms in Taiwanese populations. Our findings additionally contribute to the understanding of genetically defined PD patients successfully treated with STN-DBS.

## Abbreviations

DBS: deep brain stimulation
NGS: next generation sequencing
STN: subthalamic nucleus
UPDRS: unified Parkinson’s disease rating scale
VPS35: vacuolar protein sorting-associated protein 35
WGS: whole genome sequencing
